# Supplementary material for: Active site specificity profiling datasets of matrix metalloproteinases (MMPs) 1, 2, 3, 7, 8, 9, 12, 13 and 14
Source: Data Brief. 2016 Feb 22;7:299–310. doi: 10.1016/j.dib.2016.02.036 (PMC4777984; doi:10.1016/j.dib.2016.02.036)
Supplement: Supplementary file 10 — Supplementary material [file mmc10.zip › WebPICS_hMMP3_G_1%/subsite_coop.html]

 

PICS results


- # About
- # Manual
- # Analysis
- # Results

- Redraw
- Seqlogo
- Dependency
- Coop

**Potential subsite cooperativity analysis for hMMP3\_G\_1%**   
Minimum difference for subsite dependency was set to +- 10 percentage-points  
Subsite dependency was checked for all positional occurences above 1 x natural abundance  
**Subsite dependency check is restricted to P3 - P3'**

|  |  |  |  |
| --- | --- | --- | --- |
| Fixed residue | Affected residue(s) | Change (percentage-points) | Vice-Versa change |
| P3\_A | P1prime\_C | 13.4 | 26.8 |
| P3\_A | P1prime\_V | 11.1 | 10.6 |
| P3\_A | P2\_L | 13.7 | 16.2 |
| P3\_A | P2prime\_L | 27.4 | 28.9 |
| P3\_I | P1\_N | 45.6 | 24.7 |
| P3\_I | P1prime\_V | 24.6 | 15.2 |
| P3\_I | P2\_H | 11.4 | 24.7 |
| P3\_I | P2\_L | 34.9 | 26.7 |
| P3\_I | P2prime\_H | 12.1 | 31.4 |
| P3\_I | P2prime\_R | 14.5 | 14.5 |
| P3\_I | P2prime\_T | 16.5 | 21.4 |
| P3\_I | P3prime\_A | 21.3 | 10.6 |
| P3\_L | P2\_C | 13.0 | 31.6 |
| P3\_L | P2\_S | 14.9 | 19.5 |
| P3\_L | P2prime\_H | 14.3 | 48.7 |
| P3\_L | P2prime\_Q | 11.0 | 18.7 |
| P3\_L | P3prime\_A | 29.9 | 19.5 |
| P3\_P | P1\_N | -13.6 | -24.3 |
| P3\_P | P2\_Q | 11.6 | 35.8 |
| P3\_P | P3prime\_A | -10.2 | -17.0 |
| P3\_V | P2\_A | 20.8 | 17.9 |
| P2\_A | P1prime\_I | -10.3 | -11.5 |
| P2\_C | P1prime\_I | 39.9 | 10.8 |
| P2\_C | P2prime\_C | 26.6 | 62.1 |
| P2\_C | P2prime\_H | 39.6 | 55.4 |
| P2\_F | P1\_Q | 24.7 | 11.4 |
| P2\_F | P1prime\_Q | 26.7 | 16.0 |
| P2\_F | P1prime\_V | 52.8 | 15.0 |
| P2\_F | P2prime\_I | 38.1 | 12.7 |
| P2\_F | P3prime\_I | 25.4 | 12.7 |
| P2\_F | P3prime\_N | 42.7 | 23.3 |
| P2\_G | P1\_K | 17.1 | 24.7 |
| P2\_G | P3prime\_A | 21.3 | 10.6 |
| P2\_H | P1\_Y | 12.1 | 10.3 |
| P2\_H | P1prime\_Y | 13.4 | 16.0 |
| P2\_H | P2prime\_R | 41.4 | 19.1 |
| P2\_H | P2prime\_T | 26.7 | 16.0 |
| P2\_L | P1\_N | 19.4 | 13.7 |
| P2\_L | P1prime\_C | 16.9 | 28.7 |
| P2\_L | P2prime\_I | 17.5 | 16.5 |
| P2\_L | P2prime\_T | 16.9 | 28.7 |
| P2\_L | P3prime\_L | 12.2 | 12.2 |
| P2\_Q | P1\_A | 11.5 | 10.7 |
| P2\_Q | P1\_Y | 16.8 | 33.6 |
| P2\_S | P1\_H | 12.1 | 31.4 |
| P2\_S | P2prime\_K | 22.6 | 12.2 |
| P1\_A | P1prime\_Q | 13.4 | 20.1 |
| P1\_A | P3prime\_G | 12.7 | 17.4 |
| P1\_C | P1prime\_V | 86.1 | 12.3 |
| P1\_C | P3prime\_M | 30.0 | 18.0 |
| P1\_D | P1prime\_C | 13.4 | 20.1 |
| P1\_D | P2prime\_I | 28.1 | 23.4 |
| P1\_D | P3prime\_N | 12.7 | 17.4 |
| P1\_F | P2prime\_V | 53.5 | 16.0 |
| P1\_F | P3prime\_N | 26.0 | 14.2 |
| P1\_H | P1prime\_Y | 16.7 | 16.7 |
| P1\_H | P2prime\_Q | 33.4 | 16.7 |
| P1\_K | P2prime\_L | 43.0 | 20.3 |
| P1\_K | P3prime\_G | 48.3 | 39.5 |
| P1\_N | P1prime\_I | -13.0 | -12.1 |
| P1\_N | P3prime\_A | 24.5 | 22.6 |
| P1\_N | P3prime\_K | -12.4 | -11.9 |
| P1\_Q | P1prime\_V | 16.9 | 10.4 |
| P1\_Q | P2prime\_I | 26.6 | 19.2 |
| P1\_S | P2prime\_L | 23.1 | 17.0 |
| P1\_S | P2prime\_R | 12.8 | 13.8 |
| P1\_Y | P2prime\_C | 26.6 | 62.1 |
| P1prime\_C | P2prime\_I | 18.1 | 10.1 |
| P1prime\_C | P3prime\_L | 28.7 | 16.9 |
| P1prime\_I | P2prime\_H | 12.1 | 62.8 |
| P1prime\_I | P2prime\_L | 14.3 | 19.6 |
| P1prime\_I | P3prime\_A | 13.6 | 13.6 |
| P1prime\_I | P3prime\_G | 11.9 | 28.3 |
| P1prime\_Q | P3prime\_G | 12.7 | 11.6 |
| P1prime\_Q | P3prime\_I | 12.1 | 10.1 |
| P1prime\_V | P2prime\_I | 16.7 | 19.4 |
| P1prime\_V | P2prime\_T | 12.4 | 26.1 |
| P2prime\_H | P3prime\_A | 62.8 | 12.1 |
| P2prime\_I | P3prime\_N | 26.0 | 42.6 |
| P2prime\_L | P3prime\_G | 13.8 | 23.8 |
| P2prime\_L | P3prime\_K | 15.0 | 11.4 |
| P2prime\_Q | P3prime\_L | 28.7 | 16.9 |
| P2prime\_T | P3prime\_A | 32.8 | 12.6 |
| P2prime\_T | P3prime\_I | 12.1 | 10.1 |
| P2prime\_V | P3prime\_I | 17.1 | 28.5 |

  
**PICS analysis of protease: hMMP3\_G\_1%**  
151 cleavage sites analyzed  
PICS library made with (T)rypsin, (G)luC or (C)hymotrypsin:   
Cutoff for graphic display: 2 x natural abundance  

|  |  |
| --- | --- |
| Positional occurences  (table for total and relative (in %) values) | Occurences relative to natural abundance (table) |
|  |  |
